# Maturation and migration of murine CD4 single positive thymocytes and thymic emigrants

**DOI:** 10.5936/csbj.201403003

**Published:** 2014-03-30

**Authors:** Xi Xu, Qing Ge

**Affiliations:** a Key Laboratory of Medical Immunology, Ministry of Health. Department of Immunology, School of Basic Medical Sciences, Peking University Health Science Center, 38 Xue Yuan Road, Beijing 100191, P R China

**Keywords:** single positive thymocytes, thymic output, recent thymic emigrants

## Abstract

T lymphopoiesis in the thymus was thought to be completed once they reach the single positive (SP) stage, when they are “fully mature” and wait to be exported at random or follow a “first in-first out” manner. Recently, accumulating evidence has revealed that newly generated SP thymocytes undergo further maturation in the thymic medulla before they follow a tightly regulated emigrating process to become recent thymic emigrants (RTEs). RTEs in the periphery then experience a post-thymic maturation and peripheral tolerance and eventually become licensed as mature naïve T cells. This review summarizes the recent progress in the late stage T cell development in and outside of the thymus. The regulation of this developmental process is also discussed.

## Introduction

The thymus provides a unique microenvironment for the development and maturation of T lymphocytes. The key events during T cell development include: the entry of lymphoid progenitor cells from the bone marrow into the thymus and the differentiation into T cell precursors; the formation of functional T cell receptor (TCR) through TCR β- and α-chain rearrangement; positive and negative selection to ensure the major histocompatibility complex (MHC) restriction to self-peptide as well as clearance of autoreactive cells [1–4]. After a highly regulated developmental process in the thymus, only about 1% of the thymocytes are able to emigrate and join the peripheral lymphocyte pool [5, 6].

Compared to the early stages of thymocyte development where detailed developmental process and the underlying mechanisms are reasonably well defined, the late stage development, in particular, the development of CD4 or CD8 single positive (SP) thymocytes, the thymic egress, and the post-thymic maturation of recent thymic emigrants (RTEs) have been largely ignored. SP thymocytes were thought to be “fully mature”, and could leave the thymus at random or follow an ordered “first in-first out” manner [7]. Recent studies, however, revealed a dynamic and eventful developmental program for SP thymocytes in the thymus as well as RTEs in the periphery. The migration of these young T cells from the thymus to the periphery was also found to be tightly regulated. As the peripheral maturation of RTEs has been discussed in detail by Fink et al. [8, 9], we mainly summarize the recent progress on the maturation and emigration of SP thymocytes.

## The development of SP thymocytes in the thymus

### The migration and residence of SP thymocytes in the thymus

After positive selection, the survived CD4 and CD8 double positive (DP) thymocytes relocate from the thymic cortex to the medulla, down-regulate one of the coreceptors and become CD4 or CD8 SP thymocytes. A direct precursor-product relationship between dividing cortical DP cells and mature medullary SP thymocytes was estimated to be within 1-3 days using bromodeoxyuridine (BrdU) incorporation approach [10, 11].

The residence time of SPs in the thymic medulla, however, varies from 4 to 12 days based on different experimental settings. For instance, with continuous [^3^H] thymidine incorporation, Egerton et al. demonstrated that the complete replacement of the medullary compartment took about 12 days [12]. The results from pulse labeling with BrdU suggested that the turnover of BrdU^+^ SP thymcoytes occurred in 5-7 days [13]. Similarly, the intrathymic delivery of MHC-expressing adenoviruses into MHC class II-deficient mice led to a conclusion of 6-7-day of residence time [14]. The adoptive transfer of the earliest SP thymocyte subset also resulted in a 4-7-day persistence in the thymus before egress [15]. Despite the differences in suggested residency time, it remains to be determined how SP thymocytes are regulated to finish the maturation program and central tolerance before acquiring egress capability.

### The developmental program of SP thymocytes

With the help of many newly discovered cell surface molecules, investigators came to realize the heterogeneity of medullary SP thymocytes. For instance, based on the expression of CD24 (HSA), SP thymocytes can be divided into two subgroups, with the CD24^-^ ones being more mature than the CD24^+^ ones, producing more cytokines upon activation [16]. CD69 is expressed in only a fraction of TCR^+^ SP thymocytes [17], whereas Qa2 is expressed in SPs with more mature functions [18]. Thus, several developmental pathways have been proposed to link the phenotypic differences of SPs with their functional maturity. Take a two-stage scheme for example, cells at the early stage with a phenotype of CD69^+^CD24^+^Qa2^-^, barely responded to Con A or anti-CD3 stimulation while those at the late stage with a phenotype of CD69^-^CD24^-^Qa2^+^ responded quite well by proliferation and secretion of a variety of cytokines [13, 19–21]. A different combination of CD69 and CD62L expression revealed another two-stage scheme for SP thymocytes: CD69^+^CD62L^lo^ and CD69^-^CD62L^hi^ [22]. However, other evidence has suggested the existence of intermediate stages [23, 24].

Based on the expression of CCR7, two types of three-stage models were proposed for the studies of negative selection and nTreg development. The first one demonstrated that CD4 SP thymocytes can be divided into SP1 (CD24^+^CCR7^-^), SP2 (CD24^+^CCR7^+^), and SP3 (CD24^-^CCR7^+^) [11]. The second one combined CD69, CCR9 with CCR7, and suggested a slightly different developmental program: CD69^+^CCR7^^-^/_lo_^CCR9^+^, CD69^+^CCR7^+^CCR9^-^, and CD69^-^CCR7^+^CCR9^-^ [25]. However, no functional studies were undertaken to compare these subsets.

**Figure 1:**
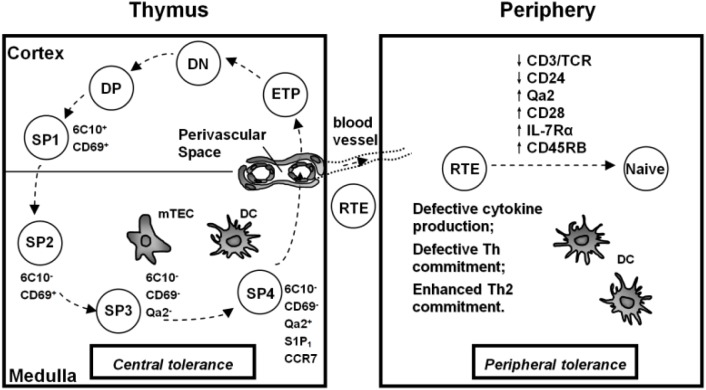
Late stage development of murine CD4 single positive T cells in and outside of the thymus. ETP, Early T lineage progenitor; DN, double negative; DP, double positive; SP, single positive; RTE, recent thymic emigrants; mTEC, medullary thymic epithelial cells; DC, dendritic cells.

Recently, we have resolved TCRαβ^+^CD4^+^CD8^-^ thymocytes into four subsets: SP1 (6C10^+^CD69^+^), SP2 (6C10^-^CD69^+^), SP3 (CD69^-^Qa2^-^), and SP4 (CD69^-^Qa2^+^) (Figure 1) [26]. The functional comparison, microarray analysis, and the sequential appearance of these subsets during mouse ontogeny and after the intrathymic adoptive transfer of SP1 cells have confirmed that these four subsets define a sequential and irreversible multistage program for the development of CD4 SP thymocytes [15, 26]. The cell proliferation and cytokine secretion upon Con A or anti-CD3 and anti-CD28 stimulation, such as IL-2, IL-4 and IFN-γ, were found to be progressively enhanced from SP1 to SP4 [15, 26]. The migration from the thymic cortex to the medulla may not occur until SP thymocytes reach SP2 stage according to the different expression of PlexinD1 and CCR7 (PlexinD1 suppresses the signaling of CCR9/CCL25 for cortex retention and CCR7 promotes the cortex-to-medulla migration of thymocytes) in SP1 and SP2 subsets [27]. Based on the expression of S1P_1_ and the phenotype of fluorescein isothiocyanate (FITC)^+^ T cells in the periphery 24 hours after FITC intrathymic injection, SP4 thymocytes were believed to be capable of emigrating from the thymus [27, 28].

When investigating the developmental program of CD4 SP thymocytes in the thymus, one important issue is the interference of T cells that have recirculated back to the thymus from the periphery. Recirculating T cells in the thymus can be detected in mice from 2 days to nearly 2 years of age [29, 30]. In young adult mice, only 1-2% of CD4 or CD8 single positive cells in the thymus are recirculating T cells. The proportion increases dramatically with age, reaching over 20% in mice nearly 2 years of age [29, 30]. Recirculating T cells are phenotypically more mature and some express activation markers when compared to newly generated SP thymocytes [29]. The role of these cells on the maturation of SP thymocytes is not clear yet. However, these cells need to be excluded when analyzing SP thymocytes. A purification strategy of selecting CD44^lo^ SP thymocytes or GFP^+^ SP thymocytes (RAG-GFP transgenic mice) could help us exclude the returning CD44^hi^ T cells and GFP^-^ mature T cells in the thymus.

### Negative selection and the thymic microenvironment

Negative selection is a process in which newly generated T cells are rendered non-reactive to self-antigens and those with strong reactivity to self-peptide-MHC complex are deleted by apoptosis. Studies in the last twenty years have revealed that negative selection mainly takes place in the thymic medulla [13, 31, 32]. Thus, defects in T cell migration towards the thymic medulla or mice with disorganized thymic medulla are often associated with an impaired negative selection and increased autoreactive T cells in the periphery. Examples of these include mice deficient in *Ccr7* [33], *Relb* [34–36], *Nfkb2* [37], *Nik* [38], lymphotoxin β receptor (*Ltbr*) [39], *Traf6* [40], or autoimmune regulator (*Aire*) [26, 41–43]. Among these molecules, CCR7 regulates the cortex-to-medulla migration of SP thymocytes, whereas the rest are all involved in thymic epithelial cell differentiation and function [33–43].

Medullary thymic epithelial cells (mTECs) and medullary dendritic cells (mDCs) are two main antigen presenting cells (APCs) in the thymus (Figure 1) [3, 44]. mTECs express a variety of tissue specific antigens (TSA) normally found in the periphery. This is partially attributed to the function of Aire [42, 45–48]. Indeed, AIRE deficiency leads to autoimmune polyendocrinopathy-candidiasisectodermal dystrophy (APECED) in human and similar organ-specific autoimmune diseases in mouse [42, 49, 50]. Increasing evidence suggests that the role of Aire is not limited to the regulation of TSA expression. Aire also affects antigen processing by mTECs as well as appropriate differentiation of mTECs [44]. NF-κB signaling plays an important role in regulating Aire expression and mTEC maturation. Thus, the perturbation of this signaling pathway, such as in mice deficient in LTβR, its ligands LTβ or Light, CD40, and Rank, results in defective negative selection and increased autoimmunity [39, 41, 51, 52].

In addition to mTECs, mDCs are also very important for negative selection. Selective depletion of thymic DC in a transgenic mouse model resulted in an increased frequency of CD4 SP thymocytes and the development of autoimmunity [53]. The processing and presentation of TSA by mDCs is derived mostly from DCs’ uptake of apoptotic mTECs [54–56]. Migratory DCs from the peripheral blood also bring peripheral antigens to the thymus, thus further promoting central tolerance [57–59].

Although it is now well accepted that negative selection takes place in the thymic medulla, it remains largely unknown at which developmental stage of SP thymocytes that negative selection starts and at which stage it ends. In *Relb* ^-/-^ mice that have defects in negative selection due to the significant reduction of mTECs and mDCs, a developmental blockage was observed between SP3 and SP4 subsets. Such developmental arrest was also revealed in *Aire* ^-/-^ mice. This suggests that the SP3/SP4 transition could be a critical checkpoint for CD4 SP thymocyte development and negative selection [26]. Interestingly, Cowan et al. reported that the development of Qa2^+^ SP thymocytes could be supported by *Relb*-deficient mTECs in the model in which a lymphoid fetal thymus organ culture from *Relb* ^-/-^ embryos was grafted into wild type mice [25]. As the evidence from our group and Fink group suggested that DCs could promote the upregulation of Qa2 in SP thymocytes and RTEs [28, 60], it is likely that wild type DCs in Cowan's model migrate to the grafted thymus and promote the phenotypic maturation of SP thymocytes. However, whether the functional maturation of CD4 SP thymocytes is impaired in this model is awaiting for further examination.

Recently, the expression of Ikaros family transcription factor Helios was used to mark the SP thymocytes that undergo negative selection. Daley et al. showed that with the coinduction of Helios and the proapoptotic protein Bim, CCR7^-^CD4^+^CD69^+^ thymocytes up-regulated PD-1, down-regulated CD4 and CD8 and underwent Bim-dependent apoptosis. On the contrary, Helios^+^CCR7^+^CD4^+^ thymocytes revealed Card11- and c-Rel–dependent activation that opposes Bim-mediated apoptosis. Such activation did not result in the proliferation of SPs due to the lack of growth mediators such as IL-2 and Myc [11]. However, the role of this “hollow” activation of autoreactive cells and the eventual fate of these Helios^+^CCR7^+^ cells remain unclear.

## Thymic output

### The overview of thymic output

After highly regulated developmental process in the thymus, only about 1% of the thymocytes are able to emigrate as RTEs and join the population of peripheral lymphocytes. By comparing the phenotype of SP thymocytes and thymic emigrants within 24 hours of egress, Dong et al. demonstrated that the main thymic population in adult mice that enters the periphery is the functionally most mature SP4 subset with a phenotype of CD69^-^Qa2^+^. In neonatal mice, however, the RTE precursors bear a phenotype of SP3 cells (CD69^-^Qa2^-^) [28]. It awaits for further investigation whether such phenotypic difference of RTEs is due to a difference in progenitors in newborn versus adult mice or unique mechanisms to promote a fast establishment of the peripheral T cell pool by premature thymic egress [61]. Despite the difference in phenotypic composition, the export ratio of RTEs to total thymocytes keeps constant during lifetime in healthy individuals, while the absolute number changes with the size of the thymus [5, 8, 29, 62]. Thus, the detection of RTEs in the periphery was often used as a marker to predict the status of thymopoiesis in patients with various diseases. Before the mechanism of egress is completely understood, it is hard, however, to use the numbers of circulating RTEs to distinguish between the impaired thymopoiesis and defective egress under certain disease states [63–65].

The export of thymocytes to the periphery is thought to occur mainly through perivascular space (PVS) (Figure 1). The thymic PVS is composed of a vascular basement membrane and a second basement membrane bordering the thymic parenchyma [66]. While some studies suggested that the PVS was located in the medulla and cortico-medullary junction of the thymus, others claimed that PVS could also be found in the thymic cortex [67–70]. The presence of lymphocytes in normal PVS has been observed by transmission electron-microscope (TEM) and scanning electron-microscope (SEM), while a giant PVS filled with thymocytes could also be found in the non-obese diabetic (NOD) mice under fluorescence microscope [69–72]. The agonist of S1P, FTY720, an immunosuppressive compound that perturbs S1P- and S1P_1_-mediated signals, inhibited the egress of thymocytes and caused the accumulation of mature thymocytes in the PVS. It suggests that thymic PVS may be the main site for thymic output [33, 73–75]. More direct evidence was revealed by intravenous injection of PE-conjugated CD4 antibody. Within 5 minutes of injection, CD4^+^ T cells in the PVS could be selectively labeled, with the great majority located within 50 µm of the cortico-medullary junction on thymic cross sections. Calculating the number of labeled cells per thymus further confirmed that the majority of thymocytes emigrate via PVS at the cortico-medullary junction [6]. Moreover, lymphatic vessels could also be involved in thymocyte emigration, though they might be more important in aged or diseased animals [76, 77].

### The regulation of thymocyte egress

The mechanisms controlling thymic output remain elusive until recently. Early studies suggested, based on relatively mature but heterogeneous phenotype of RTEs, that thymocytes leave the thymus at random or follow an ordered “first in-first out” manner [7]. Recent studies, however, revealed more complex regulation.

It was demonstrated that sphingosine-1-phosphate (S1P) and one of its receptors, S1P_1_, can regulate thymocyte emigration. S1P_1_ is a G protein coupled receptor (GPCR) that expressed in mature SP thymocytes, while S1P is produced by vascular endothelium as well as neural crest-derived pericytes that ensheathe the blood vessels [6, 78–80]. The high concentration of S1P in the blood and around PVS attracts S1P_1_-expressing mature thymocytes to export [6, 81]. The importance of S1P-S1P_1_ signaling in regulating thymocyte emigration is further evidenced by mice deficient in S1P_1_, sphingosine kinases (essential for the production of S1P) or lipid phosphate phosphatase 3 (LPP3, can degradate thymic S1P), with significantly decreased thymic output and accumulated thymocytes in the thymic medulla [6, 79, 80, 82]. Based on these data, the search for thymic RTE precursors was much easier as the expression of *S1pr1* (the gene that encodes S1P_1_) was found highest in SP4 thymocytes. The expression of *Foxo1*, the transcriptional factor that regulates the expression of *S1pr1*, also peaked at SP4 thymocytes, suggesting that CD4 SP thymocytes may not acquire the ability to egress until the cells reach SP4 stage [27]. Other factors, such as KLF2 (transcriptional factor of S1P_1_), PI3K (negative regulator of KLF2) and PTEN (negative regulator of PI3K) can also influence thymocyte emigration [83–85].

In addition to S1P-mediated chemotaxis, CCR7, another member of the GPCR family, and one of its ligands CCL19, also contribute to thymocyte emigration. CCL19 attracts mature T cells migrating out of the fetal thymus organ culture through its interaction with CCR7. Interestingly, another ligand of CCR7, CCL21, fails to show the involvement in thymic emigration [86]. The chemorepellent signals provided by thymic stroma, including the chemokine stromal-derived factor (SDF)-1 (or CXCL12), which repels T cells via a CXCR4 receptor mediated manner, might also be important for thymic emigration [87, 88].

Except for GPCR mediated thymic emigration, other factors such as early growth response gene 1 (Egr1) [89], aryl hydrocarbon receptor (AHR) [90], laminin-5 [91] and VLA-5 (integrin α5β1) [92] have all been demonstrated to be involved in the egress process. Egr1 is a transcriptional regulator whose expression can be induced by multiple signals including TCR. Egr1-deficient mice have poor accumulation of RTEs in the periphery, and this appears to originate from the decreased survival of mature thymocytes and RTEs [89]. AHR is a ligand-dependent member of the PAS-bHLH family of nuclear receptors. The overactivatoin of AHR leads to the preferential emigration of DN thymocytes and their accumulation in the spleen [90]. Laminin-5 is expressed in the thymic medulla. Interactions of thymocytes with laminin-5 induced the release of a soluble fragment of surface molecule CD44, which leads to an increased migration of medullary thymocytes [91]. On the other hand, a defective expression of VLA-5 on NOD thymocytes was found to correlate with a decreased thymic output and a giant PVS filled with mature thymocytes [92].

## Post-thymic maturation and peripheral tolerance of RTEs

### The dynamics and homeostatic properties of RTEs

The idea that T cell development occurred only in the thymus was widely accepted until 1970s, when it was first proposed that T cells left the thymus in an immature state and completed their development in the periphery [93]. Subsequent studies further revealed that RTEs and peripheral mature naïve T cells are different both phenotypically and functionally [8, 94]. The uniqueness of RTEs as a subpopulation different from SP thymocytes as well as mature naïve T cells has been gradually realized [8, 94–99].

The proportion of RTEs to peripheral T cells changes during lifetime. In mice, RTEs occupy the entire peripheral T cell pool at 1-3 weeks of age. The ratio drops to 20% in young adult period, and further declines to 3% in 6-months old mice. But RTEs can be clearly detected in mice reaching 2-year of age [29]. RTEs are widely distributed in the periphery, as studies have found RTEs in the lymph nodes, Peyer's patches, spleen, blood and small intestine of mice [94, 95, 100]. Since RTEs and peripheral naïve T cells occupy some overlapping areas, researchers are eager to know which ones have the survival advantage over the other. Berzins et al. and Dong et al. reported that RTEs were preferentially incorporated into the peripheral T cell pool at the expense of their mature naive T cell counterparts. In contrast, Houston et al. found that RTEs preferentially accumulated only in a lymphopenic environment due to their higher expression of CD24. In lymphoreplete mice, RTEs were disadvantaged competitors as both RTEs and naïve T cells had to compete for a limited survival niche [28, 100–102]. Such discrepancy may come from different methods used to perform the experiments. Berzins et al. studied RTEs from hyperthymic (thymus-grafted) mice, whereas Dong et al. and Houston et al. performed adoptive transfer experiments with different subpopulations. Dong et al. compared the survival of CD4^+^ RTEs precursors (Qa2^+^CD69^-^ SP4 thymocytes) and CD4^+^ naïve T cells, and found that CD4^+^ pre-RTEs had the survival advantage over CD4^+^ naïve T cells in the periphery, while Houston et al. used CD4^+^ RTEs from the lymph nodes in the comparison and arrived at a different conclusion [28, 100, 101]. Thus, it is reasonable to think that RTEs purified from the lymph nodes have received maturation signals from the periphery and changed their homeostatic properties from their precursors in the thymus.

### Methods in RTE studies

To facilitate the study of RTEs, several methods have been developed to distinguish them from other peripheral T cells. One method, direct intrathymic injection of FITC, can efficiently label murine thymocytes, enabling their subsequent identification in the peripheral lymphoid tissues. The advantage of this method is a direct phenotypic and functional study of the youngest RTEs by the analysis of peripheral FITC^+^ T cells. However, the surgical stress to the animals, limitied time frame for detection (usually within 24 hours) as well as non-specific labeling of mature T cells recirculating to the thymus limit the wide application of this method [8, 103, 104]. The second method is giving the animals BrdU. Since BrdU is taken up by dividing thymocytes, RTEs have been identified as BrdU^lo^. However, this population is contaminated with post-division BrdU^lo^ mature T cells, blurring the distinction between RTEs and older peripheral T cells. Furthermore, detection of BrdU incorporation precludes functional studies [8, 105]. The third method relies on T cell receptor rearrangement excision circles (TRECs). TRECs are stable and nonreplicative extrachromosomal circles of excised DNA during TCR gene recombination and are enriched in RTEs. However, not all TREC^+^ cells are RTEs since they can still be detected in the periphery after thymectomy; not all RTEs are TREC^+^ because only one daughter cell can get the excised DNA in division. Moreover, the TREC analysis is usually performed by real-time PCR, precluding the further phenotypic and functional characterization of this specific cell population [106–109]. Other methods such as thymic lobe grafts and fetal thymus organ culture were also used but they all have limitations such as creating an artificial full peripheral T cell compartment or introducing an in vitro system that may not accurately reflect the in vivo environment [5, 8, 96, 100]. Some cell surface markers were also tested to define RTEs. For instance, mouse RTEs were defined as Qa2^lo^CD24^hi^ and human RTEs were defined as CD31^+^PTK7^+^. Although this method allows for readily phenotypic and functional analysis of cells, whether these markers define the majority of peripheral RTEs was questioned [94, 110, 111].

Recently, the development of RAG1-GFP knockin and RAG2p-GFP transgenic mice made a big progress in RTE research. In these mice, the expression of GFP is driven by RAG1 or RAG2 promoter. After RAG gene expression is extinguished, the GFP signal remains detectable for a few more weeks, enabling the tracking of RTEs by GFP^+^ peripheral T cells [8, 94]. According to the half-life of GFP protein, GFP^hi^ peripheral T cells have left the thymus within a week, GFP^lo^ ones are 1–2 weeks older and GFP^–^ cells represent the mature peripheral T cells [8, 94]. This method allows the studies of live RTEs from unmanipulated mice. The residence time of RTEs in the periphery can be also indicated by the intensity of GFP signal. However, the intensity of GFP signal can be diluted by cell division. More importantly, even GFP^hi^ RTEs may have stayed in the periphery for a few days and may have received some regulation and made necessary alterations in the secondary lymphoid organs. It is thus hard to predict the immediate changes after thymic egress [8, 94]. Despite these disadvantages, the RAG-GFP mice have been widely used in RTE research.

### Post-thymic maturation of RTEs

RTEs are immature compared to peripheral naïve T cells, rendering post-thymic maturation necessary both phenotypically and functionally (Figure 1). Using RAG2p-GFP transgenic mice, the phenotypic analysis demonstrated that during their transition from GFP^hi^ to GFP^lo^ and finally GFP^-^, RTEs gradually down-regulated CD24 and CD3/TCR, and up-regulated Qa2, CD28, CD45RB, and IL-7Rα [94, 96, 97]. Such pattern of phenotypic changes was supported by FITC and BrdU labeling of RTEs [20, 97, 105]. The changes of other surface markers during the maturation of RTEs include the upregulation of Ly6C, and higher levels of α4β7, αE integrin and CCR9 expression in CD8^+^ RTEs than CD8^+^ naïve T cells [95, 98, 112, 113].

In accordance with phenotypic differences, RTEs and naïve T cells are functionally distinct. Under non-polarizing conditions, activated CD4^+^ RTEs showed diminished proliferation when compared with CD4^+^ naïve T cells. This defect was only partly corrected by the addition of exogenous IL-2. Activated RTEs also secreted less IL-2, IL-4 and IFN-γ, expressed lower level of CD25 (IL-2 receptor α-chain) but similar level of CD69 [94, 114, 115]. The defect in IL-2 production was more obvious in aged mice [116]. Under Th1, Th17 and iTreg inducing conditions, RTEs expressed less characteristic cytokines or major transcription factors. On the contrary, more IL-4, IL-5 and IL-13 were produced both in vivo and in vitro in Th2-polarized RTEs when compared with Th2-polarized naïve T cells [99, 115, 117]. The ex vivo analysis of transcription factor and cytokine receptor expression suggested that instead of biased towards the Th2 cell lineage, CD4^+^ RTEs are biased away from the Th1 cell lineage [115]. Compared with CD8^+^ naïve T cells, CD8^+^ RTEs contain a lower frequency of cytolytic precursors, suggesting the existence of functional defects. Indeed, CD8^+^ RTEs secreted less tumor necrosis factor (TNF) with anti-CD3 and anti-CD28 stimulation in vitro [118]. After bacterial or viral infections, activated CD8^+^ RTEs produced less cytokines and generated fewer IL-7Rα^hi^KLRG1^lo^ memory precursor effector cells [119, 120].

The phenotypic and functional maturation process is a result of maturation at the single cell level, not selective survival and proliferation of a small population of relatively mature RTEs [60]. This leads investigators to explore the mechanisms of post-thymic maturation. First, thymic egress is required for the acquisition of a complete phenotypic maturation because mice treated with blocking antibodies such as AAL-R (a synthetic mimetic of S1P) to inhibit thymic egress showed an immature phenotype of RTE candidates [60]. Second, the phenotypic and functional maturation also requires the access to secondary lymphoid organs, as a combination of splenectomy and administration of anti-CD62L plus anti-VLA-4 to block lymph node entry also impaired RTE maturation [60]. Further studies revealed that a full dendritic cell compartment in the secondary lymphoid organs was indispensable for phenotypic maturation of RTEs while self-peptide-MHC complexes and IL-7 were dispensable [121]. The transcriptional repressor NKAP may also influence RTE maturation, as NKAP deficiency keeps RTEs from full maturation [122]. However, up to now, the mechanism of RTE maturation at the molecular level remains largely unknown.

### Peripheral tolerance of RTEs

Although central tolerance can eliminate most of the autoreactive T cells in the thymus, some of them inevitably escape and export to the periphery, rendering the necessity of peripheral tolerance to these RTEs [123, 124]. Complementarity determining region 3 (CDR3) length spectratyping revealed that TCRs expressed by RTEs were skewed toward longer CDR3 regions compared with naive T cells, suggesting the existence of more autoreactive cells in the RTE population [125–127]. The mechanisms of peripheral tolerance of RTEs are obscure with scattered evidence revealing some possible explanations. CD8^+^ RTEs could enter neonatal nonlymphoid tissues such as skin and become tolerized to antigens expressed there [128]. Higher level of α4β7, αE integrin, and CCR9 on CD8^+^ RTEs facilitated their homing to the gut-associated lymphoid tissues. This pattern of migration may allow intestine-homing RTEs to gain tolerance to self-antigens and harmless food antigens there [95, 98, 111, 113]. Moreover, RTEs appeared to be tolerized in vivo by alloantigen, as RTEs failed to cause graft-versus-host disease (GVHD) when transferred to allogeneic mice [96]. The diminished proliferation and defective cytokine secretion may contribute to their peripheral tolerance [8, 94]. Except for selective migration and immunoincompetence of RTEs mentioned above, self-antigens presented by DCs and lymphoid stroma in the periphery may also promote peripheral tolerance [129, 130]. Another important mechanism is the expression of some inhibitory receptors by RTEs, such as CTLA-4 and PD-1. Severe autoimmune disease was found in Rag^-/-^ mice when transferred with RTEs deficient in PD-1 [96, 131].

## Summary and Outlook

Previous studies have suggested that instead of simply waiting for export to the periphery, SP thymocytes undergo multiple stages of maturation in and outside of the thymus before they become mature naïve T cells. This process is under precise regulation of thymic and peripheral microenvironment. As RTEs were found enriched in several disease tissues, such as ulcerative colitis and chronic myeloid leukaemia [132, 133], it is reasonable to hypothesize that these diseases may affect the maturation of SP/RTEs and defective RTEs may play a pathological role in disease progression. Compared to our knowledge in the early stage of thymocyte development, the process of SP/RTE/Naïve T cell transition is far from being well understood. Its underlying mechanisms and its relationship to diseases should be further explored.

## References

[1] Nitta T, Murata S, Ueno T, Tanaka K, Takahama Y (2008) Thymic microenvironments for T-cell repertoire formation. Adv Immunol 99: 59–94 19117532 10.1016/S0065-2776(08)00603-2

[2] Rothenberg EV, Taghon T (2005) Molecular genetics of T cell development. Annu Rev Immunol 23: 601–649 15771582 10.1146/annurev.immunol.23.021704.115737

[3] Takahama Y (2006) Journey through the thymus: stromal guides for T-cell development and selection. Nat Rev Immunol 6: 127–135 16491137 10.1038/nri1781

[4] de Pooter R, Zuniga-Pflucker JC (2007) T-cell potential and development in vitro: the OP9-DL1 approach. Curr Opin Immunol 19: 163–168 17303399 10.1016/j.coi.2007.02.011

[5] Berzins SP, Godfrey DI, Miller JF, Boyd RL (1999) A central role for thymic emigrants in peripheral T cell homeostasis. Proc Natl Acad Sci U S A 96: 9787–9791 10449772 10.1073/pnas.96.17.9787 PMC22288

[6] Zachariah MA, Cyster JG (2010) Neural crest-derived pericytes promote egress of mature thymocytes at the corticomedullary junction. Science 328: 1129–1135 20413455 10.1126/science.1188222 PMC3107339

[7] Scollay R, Godfrey DI (1995) Thymic emigration: conveyor belts or lucky dips?. Immunol Today 16: 268–273; discussion 273-264. 7662096 10.1016/0167-5699(95)80179-0

[8] Fink PJ, Hendricks DW (2011) Post-thymic maturation: young T cells assert their individuality. Nat Rev Immunol 11: 544–549 21779032 10.1038/nri3028 PMC3241610

[9] Fink PJ (2013) The biology of recent thymic emigrants. Annu Rev Immunol 31: 31–50 23121398 10.1146/annurev-immunol-032712-100010

[10] Penit C (1986) In vivo thymocyte maturation. BUdR labeling of cycling thymocytes and phenotypic analysis of their progeny support the single lineage model. J Immunol 137: 2115–2121 3093565

[11] Daley SR, Hu DY, Goodnow CC (2013) Helios marks strongly autoreactive CD4+ T cells in two major waves of thymic deletion distinguished by induction of PD-1 or NF-kappaB. J Exp Med 210: 269–285 23337809 10.1084/jem.20121458 PMC3570102

[12] Egerton M, Scollay R, Shortman K (1990) Kinetics of mature T-cell development in the thymus. Proc Natl Acad Sci U S A 87: 2579–2582 2138780 10.1073/pnas.87.7.2579 PMC53733

[13] Lucas B, Vasseur F, Penit C (1994) Production, selection, and maturation of thymocytes with high surface density of TCR. J Immunol 153: 53–62 8207255

[14] Rooke R, Waltzinger C, Benoist C, Mathis D (1997) Targeted complementation of MHC class II deficiency by intrathymic delivery of recombinant adenoviruses. Immunity 7: 123–134 9252125 10.1016/s1074-7613(00)80515-4

[15] Jin R, Wang W, Yao JY, Zhou YB, Qian XP, et al. (2008) Characterization of the in vivo dynamics of medullary CD4 + CD8- thymocyte development. J Immunol 180: 2256–2263 18250433 10.4049/jimmunol.180.4.2256

[16] Wilson A, Day LM, Scollay R, Shortman K (1988) Subpopulations of mature murine thymocytes: properties of CD4-CD8+ and CD4 + CD8- thymocytes lacking the heat-stable antigen. Cell Immunol 117: 312–326 2973844 10.1016/0008-8749(88)90121-9

[17] Yamashita I, Nagata T, Tada T, Nakayama T (1993) CD69 cell surface expression identifies developing thymocytes which audition for T cell antigen receptor-mediated positive selection. Int Immunol 5: 1139–1150 7902130 10.1093/intimm/5.9.1139

[18] Vernachio J, Li M, Donnenberg AD, Soloski MJ (1989) Qa-2 expression in the adult murine thymus. A unique marker for a mature thymic subset. J Immunol 142: 48–56 2642507

[19] Ramsdell F, Jenkins M, Dinh Q, Fowlkes BJ (1991) The majority of CD4 + 8- thymocytes are functionally immature. J Immunol 147: 1779–1785 1679836

[20] Gabor MJ, Godfrey DI, Scollay R (1997) Recent thymic emigrants are distinct from most medullary thymocytes. Eur J Immunol 27: 2010–2015 9295039 10.1002/eji.1830270827

[21] Hayakawa K, Lin BT, Hardy RR (1992) Murine thymic CD4+ T cell subsets: a subset (Thy0) that secretes diverse cytokines and overexpresses the V beta 8 T cell receptor gene family. J Exp Med 176: 269–274 1351921 10.1084/jem.176.1.269 PMC2119274

[22] Fritsch Fredin M, Elgbratt K, Svensson D, Jansson L, Melgar S, et al. (2007) Dextran sulfate sodium-induced colitis generates a transient thymic involution--impact on thymocyte subsets. Scand J Immunol 65: 421–429 17444952 10.1111/j.1365-3083.2007.01923.x

[23] Ge Q, Chen WF (1999) Phenotypic identification of the subgroups of murine T-cell receptor alphabeta+ CD4+ CD8- thymocytes and its implication in the late stage of thymocyte development. Immunology 97: 665–671 10457221 10.1046/j.1365-2567.1999.00816.x PMC2326876

[24] Tian T, Zhang J, Gao L, Qian XP, Chen WF (2001) Heterogeneity within medullary-type TCRalphabeta(+)CD3(+)CD4(-)CD8(+) thymocytes in normal mouse thymus. Int Immunol 13: 313–320 11222500 10.1093/intimm/13.3.313

[25] Cowan JE, Parnell SM, Nakamura K, Caamano JH, Lane PJ, et al. (2013) The thymic medulla is required for Foxp3+ regulatory but not conventional CD4+ thymocyte development. J Exp Med 210: 675–681 23530124 10.1084/jem.20122070 PMC3620359

[26] Li J, Li Y, Yao JY, Jin R, Zhu MZ, et al. (2007) Developmental pathway of CD4 + CD8- medullary thymocytes during mouse ontogeny and its defect in Aire-/- mice. Proc Natl Acad Sci U S A 104: 18175–18180 17984055 10.1073/pnas.0708884104 PMC2084316

[27] Teng F, Zhou Y, Jin R, Chen Y, Pei X, et al. (2011) The molecular signature underlying the thymic migration and maturation of TCRalphabeta+ CD4+ CD8 thymocytes. PLoS One 6: e25567 22022412 10.1371/journal.pone.0025567 PMC3192722

[28] Dong J, Chen Y, Xu X, Jin R, Teng F, et al. (2013) Homeostatic properties and phenotypic maturation of murine CD4+ pre-thymic emigrants in the thymus. PLoS One 8: e56378 23409179 10.1371/journal.pone.0056378 PMC3569422

[29] Hale JS, Boursalian TE, Turk GL, Fink PJ (2006) Thymic output in aged mice. Proc Natl Acad Sci U S A 103: 8447–8452 16717190 10.1073/pnas.0601040103 PMC1482512

[30] Hale JS, Fink PJ (2009) Back to the thymus: peripheral T cells come home. Immunol Cell Biol 87: 58–64 19030016 10.1038/icb.2008.87 PMC2679673

[31] Surh CD, Sprent J (1994) T-cell apoptosis detected in situ during positive and negative selection in the thymus. Nature 372: 100–103 7969401 10.1038/372100a0

[32] Sprent J, Kishimoto H (2002) The thymus and negative selection. Immunol Rev 185: 126–135 12190927 10.1034/j.1600-065x.2002.18512.x

[33] Kurobe H, Liu C, Ueno T, Saito F, Ohigashi I, et al. (2006) CCR7-dependent cortex-to-medulla migration of positively selected thymocytes is essential for establishing central tolerance. Immunity 24: 165–177 16473829 10.1016/j.immuni.2005.12.011

[34] Weih F, Carrasco D, Durham SK, Barton DS, Rizzo CA, et al. (1995) Multiorgan inflammation and hematopoietic abnormalities in mice with a targeted disruption of RelB, a member of the NF-kappa B/Rel family. Cell 80: 331–340 7834753 10.1016/0092-8674(95)90416-6

[35] Burkly L, Hession C, Ogata L, Reilly C, Marconi LA, et al. (1995) Expression of relB is required for the development of thymic medulla and dendritic cells. Nature 373: 531–536 7845467 10.1038/373531a0

[36] Heino M, Peterson P, Sillanpaa N, Guerin S, Wu L, et al. (2000) RNA and protein expression of the murine autoimmune regulator gene (Aire) in normal, RelB-deficient and in NOD mouse. Eur J Immunol 30: 1884–1893 10940877 10.1002/1521-4141(200007)30:7<1884::AID-IMMU1884>3.0.CO;2-P

[37] Zhu M, Chin RK, Christiansen PA, Lo JC, Liu X, et al. (2006) NF-kappaB2 is required for the establishment of central tolerance through an Aire-dependent pathway. J Clin Invest 116: 2964–2971 17039258 10.1172/JCI28326 PMC1592546

[38] Kajiura F, Sun S, Nomura T, Izumi K, Ueno T, et al. (2004) NF-kappa B-inducing kinase establishes self-tolerance in a thymic stroma-dependent manner. J Immunol 172: 2067–2075 14764671 10.4049/jimmunol.172.4.2067

[39] Boehm T, Scheu S, Pfeffer K, Bleul CC (2003) Thymic medullary epithelial cell differentiation, thymocyte emigration, and the control of autoimmunity require lympho-epithelial cross talk via LtbetaR. J Exp Med 198: 757–769 12953095 10.1084/jem.20030794 PMC2194183

[40] Akiyama T, Maeda S, Yamane S, Ogino K, Kasai M, et al. (2005) Dependence of self-tolerance on TRAF6-directed development of thymic stroma. Science 308: 248–251 15705807 10.1126/science.1105677

[41] Chin RK, Lo JC, Kim O, Blink SE, Christiansen PA, et al. (2003) Lymphotoxin pathway directs thymic Aire expression. Nat Immunol 4: 1121–1127 14517552 10.1038/ni982

[42] Anderson MS, Venanzi ES, Klein L, Chen Z, Berzins SP, et al. (2002) Projection of an immunological self shadow within the thymus by the aire protein. Science 298: 1395–1401 12376594 10.1126/science.1075958

[43] Liston A, Lesage S, Wilson J, Peltonen L, Goodnow CC (2003) Aire regulates negative selection of organ-specific T cells. Nat Immunol 4: 350–354 12612579 10.1038/ni906

[44] Kroger CJ, Flores RR, Morillon M, Wang B, Tisch R (2010) Dysregulation of thymic clonal deletion and the escape of autoreactive T cells. Arch Immunol Ther Exp (Warsz) 58: 449–457 20872284 10.1007/s00005-010-0100-3

[45] Derbinski J, Schulte A, Kyewski B, Klein L (2001) Promiscuous gene expression in medullary thymic epithelial cells mirrors the peripheral self. Nat Immunol 2: 1032–1039 11600886 10.1038/ni723

[46] Gabler J, Arnold J, Kyewski B (2007) Promiscuous gene expression and the developmental dynamics of medullary thymic epithelial cells. Eur J Immunol 37: 3363–3372 18000951 10.1002/eji.200737131

[47] Kyewski B, Peterson P (2010) Aire, master of many trades. Cell 140: 24–26 20085700 10.1016/j.cell.2009.12.036

[48] Mathis D, Benoist C (2009) Aire. Annu Rev Immunol 27: 287–312 19302042 10.1146/annurev.immunol.25.022106.141532

[49] Consortium F-GA (1997) An autoimmune disease, APECED, caused by mutations in a novel gene featuring two PHD-type zinc-finger domains. Nat Genet 17: 399–403 9398840 10.1038/ng1297-399

[50] Kuroda N, Mitani T, Takeda N, Ishimaru N, Arakaki R, et al. (2005) Development of autoimmunity against transcriptionally unrepressed target antigen in the thymus of Aire-deficient mice. J Immunol 174: 1862–1870 15699112 10.4049/jimmunol.174.4.1862

[51] Derbinski J, Kyewski B (2005) Linking signalling pathways, thymic stroma integrity and autoimmunity. Trends Immunol 26: 503–506 16039157 10.1016/j.it.2005.07.006

[52] Tykocinski LO, Sinemus A, Kyewski B (2008) The thymus medulla slowly yields its secrets. Ann N Y Acad Sci 1143: 105–122 19076347 10.1196/annals.1443.018

[53] Ohnmacht C, Pullner A, King SB, Drexler I, Meier S, et al. (2009) Constitutive ablation of dendritic cells breaks self-tolerance of CD4 T cells and results in spontaneous fatal autoimmunity. J Exp Med 206: 549–559 19237601 10.1084/jem.20082394 PMC2699126

[54] Klein L, Hinterberger M, Wirnsberger G, Kyewski B (2009) Antigen presentation in the thymus for positive selection and central tolerance induction. Nat Rev Immunol 9: 833–844 19935803 10.1038/nri2669

[55] Koble C, Kyewski B (2009) The thymic medulla: a unique microenvironment for intercellular self-antigen transfer. J Exp Med 206: 1505–1513 19564355 10.1084/jem.20082449 PMC2715082

[56] Millet V, Naquet P, Guinamard RR (2008) Intercellular MHC transfer between thymic epithelial and dendritic cells. Eur J Immunol 38: 1257–1263 18412162 10.1002/eji.200737982

[57] Donskoy E, Goldschneider I (2003) Two developmentally distinct populations of dendritic cells inhabit the adult mouse thymus: demonstration by differential importation of hematogenous precursors under steady state conditions. J Immunol 170: 3514–3521 12646612 10.4049/jimmunol.170.7.3514

[58] Li J, Park J, Foss D, Goldschneider I (2009) Thymus-homing peripheral dendritic cells constitute two of the three major subsets of dendritic cells in the steady-state thymus. J Exp Med 206: 607–622 19273629 10.1084/jem.20082232 PMC2699131

[59] Hadeiba H, Lahl K, Edalati A, Oderup C, Habtezion A, et al. (2012) Plasmacytoid dendritic cells transport peripheral antigens to the thymus to promote central tolerance. Immunity 36: 438–450 22444632 10.1016/j.immuni.2012.01.017 PMC3315699

[60] Houston EG Jr, Nechanitzky R, Fink PJ (2008) Cutting edge: Contact with secondary lymphoid organs drives postthymic T cell maturation. J Immunol 181: 5213–5217 18832674 10.4049/jimmunol.181.8.5213 PMC2679686

[61] Havran WL, Allison JP (1988) Developmentally ordered appearance of thymocytes expressing different T-cell antigen receptors. Nature 335: 443–445 2458531 10.1038/335443a0

[62] Jin R, Zhang J, Chen W (2006) Thymic output: influence factors and molecular mechanism. Cell Mol Immunol 3: 341–350 17092431

[63] Jansson A, Pernestig AK, Nilsson P, Jirstrand M, Hultgren Hornquist E (2013) Toward quantifying the thymic dysfunctional state in mouse models of inflammatory bowel disease. Inflamm Bowel Dis 19: 881–888 23448795 10.1097/MIB.0b013e3182802c58

[64] Elgbratt K, Jansson A, Hultgren-Hornquist E (2012) A quantitative study of the mechanisms behind thymic atrophy in Galphai2-deficient mice during colitis development. PLoS One 7: e36726 22590596 10.1371/journal.pone.0036726 PMC3349706

[65] Elgbratt K, Bjursten M, Willen R, Bland PW, Hornquist EH (2007) Aberrant T-cell ontogeny and defective thymocyte and colonic T-cell chemotactic migration in colitis-prone Galphai2-deficient mice. Immunology 122: 199–209 17490434 10.1111/j.1365-2567.2007.02629.x PMC2265997

[66] Mori K, Itoi M, Tsukamoto N, Kubo H, Amagai T (2007) The perivascular space as a path of hematopoietic progenitor cells and mature T cells between the blood circulation and the thymic parenchyma. Int Immunol 19: 745–753 17493961 10.1093/intimm/dxm041

[67] Sainte-Marie G, Leblond CP (1964) Cytologic Features and Cellular Migration in the Cortex and Medulla of Thymus in the Young Adult Rat. Blood 23: 275–299 14130441

[68] Kostowiecki M (1967) Development of the so-called double-walled blood vessels of the thymus. Z Mikrosk Anat Forsch 77: 406–431 5629212

[69] Ushiki T, Takeda M (1997) Three-dimensional ultrastructure of the perivascular space in the rat thymus. Arch Histol Cytol 60: 89–99 9161692 10.1679/aohc.60.89

[70] Henry L, Durrant TE, Anderson G (1992) Pericapillary collagen in the human thymus: implications for the concept of the 'blood-thymus' barrier. J Anat 181(Pt 1), 39–46 1294569 PMC1259750

[71] Kato S, Schoefl GI (1989) Microvasculature of normal and involuted mouse thymus. Light- and electron-microscopic study. Acta Anat (Basel) 135: 1–11 2750455 10.1159/000146715

[72] Savino W, Carnaud C, Luan JJ, Bach JF, Dardenne M (1993) Characterization of the extracellular matrix-containing giant perivascular spaces in the NOD mouse thymus. Diabetes 42: 134–140 8093603 10.2337/diab.42.1.134

[73] Rosen H, Alfonso C, Surh CD, McHeyzer-Williams MG (2003) Rapid induction of medullary thymocyte phenotypic maturation and egress inhibition by nanomolar sphingosine 1-phosphate receptor agonist. Proc Natl Acad Sci U S A 100: 10907–10912 12954982 10.1073/pnas.1832725100 PMC196901

[74] Alfonso C, McHeyzer-Williams MG, Rosen H (2006) CD69 down-modulation and inhibition of thymic egress by short- and long-term selective chemical agonism of sphingosine 1-phosphate receptors. Eur J Immunol 36: 149–159 16342326 10.1002/eji.200535127

[75] Yagi H, Kamba R, Chiba K, Soga H, Yaguchi K, et al. (2000) Immunosuppressant FTY720 inhibits thymocyte emigration. Eur J Immunol 30: 1435–1444 10820391 10.1002/(SICI)1521-4141(200005)30:5<1435::AID-IMMU1435>3.0.CO;2-O

[76] Kato S (1997) Thymic microvascular system. Microsc Res Tech 38: 287–299 9264340 10.1002/(SICI)1097-0029(19970801)38:3<287::AID-JEMT9>3.0.CO;2-J

[77] Ji RC, Kurihara K, Kato S (2006) Lymphatic vascular endothelial hyaluronan receptor (LYVE)-1- and CCL21-positive lymphatic compartments in the diabetic thymus. Anat Sci Int 81: 201–209 17176958 10.1111/j.1447-073X.2006.00145.x

[78] Venkataraman K, Lee YM, Michaud J, Thangada S, Ai Y, et al. (2008) Vascular endothelium as a contributor of plasma sphingosine 1-phosphate. Circ Res 102: 669–676 18258856 10.1161/CIRCRESAHA.107.165845 PMC2659392

[79] Allende ML, Dreier JL, Mandala S, Proia RL (2004) Expression of the sphingosine 1-phosphate receptor, S1P1, on T-cells controls thymic emigration. J Biol Chem 279: 15396–15401 14732704 10.1074/jbc.M314291200

[80] Matloubian M, Lo CG, Cinamon G, Lesneski MJ, Xu Y, et al. (2004) Lymphocyte egress from thymus and peripheral lymphoid organs is dependent on S1P receptor 1. Nature 427: 355–360 14737169 10.1038/nature02284

[81] Schwab SR, Pereira JP, Matloubian M, Xu Y, Huang Y, et al. (2005) Lymphocyte sequestration through S1P lyase inhibition and disruption of S1P gradients. Science 309: 1735–1739 16151014 10.1126/science.1113640

[82] Breart B, Ramos-Perez WD, Mendoza A, Salous AK, Gobert M, et al. (2011) Lipid phosphate phosphatase 3 enables efficient thymic egress. J Exp Med 208: 1267–1278 21576386 10.1084/jem.20102551 PMC3173249

[83] Carlson CM, Endrizzi BT, Wu J, Ding X, Weinreich MA, et al. (2006) Kruppel-like factor 2 regulates thymocyte and T-cell migration. Nature 442: 299–302 16855590 10.1038/nature04882

[84] Sinclair LV, Finlay D, Feijoo C, Cornish GH, Gray A, et al. (2008) Phosphatidylinositol-3-OH kinase and nutrient-sensing mTOR pathways control T lymphocyte trafficking. Nat Immunol 9: 513–521 18391955 10.1038/ni.1603 PMC2857321

[85] Barbee SD, Alberola-Ila J (2005) Phosphatidylinositol 3-kinase regulates thymic exit. J Immunol 174: 1230–1238 15661877 10.4049/jimmunol.174.3.1230

[86] Ueno T, Hara K, Willis MS, Malin MA, Hopken UE, et al. (2002) Role for CCR7 ligands in the emigration of newly generated T lymphocytes from the neonatal thymus. Immunity 16: 205–218 11869682 10.1016/s1074-7613(02)00267-4

[87] Poznansky MC, Olszak IT, Evans RH, Wang Z, Foxall RB, et al. (2002) Thymocyte emigration is mediated by active movement away from stroma-derived factors. J Clin Invest 109: 1101–1110 11956248 10.1172/JCI13853 PMC150941

[88] Poznansky MC, Olszak IT, Foxall R, Evans RH, Luster AD, et al. (2000) Active movement of T cells away from a chemokine. Nat Med 6: 543–548 10802710 10.1038/75022

[89] Schnell FJ, Kersh GJ (2005) Control of recent thymic emigrant survival by positive selection signals and early growth response gene 1. J Immunol 175: 2270–2277 16081795 10.4049/jimmunol.175.4.2270

[90] Temchura VV, Frericks M, Nacken W, Esser C (2005) Role of the aryl hydrocarbon receptor in thymocyte emigration in vivo. Eur J Immunol 35: 2738–2747 16114106 10.1002/eji.200425641

[91] Vivinus-Nebot M, Rousselle P, Breittmayer JP, Cenciarini C, Berrih-Aknin S, et al. (2004) Mature human thymocytes migrate on laminin-5 with activation of metalloproteinase-14 and cleavage of CD44. J Immunol 172: 1397–1406 14734715 10.4049/jimmunol.172.3.1397

[92] Cotta-de-Almeida V, Villa-Verde DM, Lepault F, Pleau JM, Dardenne M, et al. (2004) Impaired migration of NOD mouse thymocytes: a fibronectin receptor-related defect. Eur J Immunol 34: 1578–1587 15162427 10.1002/eji.200324765

[93] Stutman O (1978) Intrathymic and extrathymic T cell maturation. Immunol Rev 42: 138–184 32648 10.1111/j.1600-065x.1978.tb00261.x

[94] Boursalian TE, Golob J, Soper DM, Cooper CJ, Fink PJ (2004) Continued maturation of thymic emigrants in the periphery. Nat Immunol 5: 418–425 14991052 10.1038/ni1049

[95] Staton TL, Habtezion A, Winslow MM, Sato T, Love PE, et al. (2006) CD8+ recent thymic emigrants home to and efficiently repopulate the small intestine epithelium. Nat Immunol 7: 482–488 16582913 10.1038/ni1319

[96] Lee CK, Kim K, Welniak LA, Murphy WJ, Muegge K, et al. (2001) Thymic emigrants isolated by a new method possess unique phenotypic and functional properties. Blood 97: 1360–1369 11222381 10.1182/blood.v97.5.1360

[97] Kelly KA, Scollay R (1990) Analysis of recent thymic emigrants with subset- and maturity-related markers. Int Immunol 2: 419–425 2085486 10.1093/intimm/2.5.419

[98] Svensson M, Marsal J, Ericsson A, Carramolino L, Broden T, et al. (2002) CCL25 mediates the localization of recently activated CD8alphabeta(+) lymphocytes to the small-intestinal mucosa. J Clin Invest 110: 1113–1121 12393847 10.1172/JCI15988 PMC150799

[99] Opiela SJ, Koru-Sengul T, Adkins B (2009) Murine neonatal recent thymic emigrants are phenotypically and functionally distinct from adult recent thymic emigrants. Blood 113: 5635–5643 19168791 10.1182/blood-2008-08-173658 PMC2689058

[100] Berzins SP, Boyd RL, Miller JF (1998) The role of the thymus and recent thymic migrants in the maintenance of the adult peripheral lymphocyte pool. J Exp Med 187: 1839–1848 9607924 10.1084/jem.187.11.1839 PMC2212318

[101] Houston EG Jr, Higdon LE, Fink PJ (2011) Recent thymic emigrants are preferentially incorporated only into the depleted T-cell pool. Proc Natl Acad Sci U S A 108: 5366–5371 21402911 10.1073/pnas.1015286108 PMC3069187

[102] Li O, Zheng P, Liu Y (2004) CD24 expression on T cells is required for optimal T cell proliferation in lymphopenic host. J Exp Med 200: 1083–1089 15477346 10.1084/jem.20040779 PMC2211842

[103] Butcher EC, Weissman IL (1980) Direct fluorescent labeling of cells with fluorescein or rhodamine isothiocyanate. I. Technical aspects. J Immunol Methods 37: 97–108 7003013 10.1016/0022-1759(80)90195-7

[104] Scollay RG, Butcher EC, Weissman IL (1980) Thymus cell migration. Quantitative aspects of cellular traffic from the thymus to the periphery in mice. Eur J Immunol 10: 210–218 7379836 10.1002/eji.1830100310

[105] Tough DF, Sprent J (1994) Turnover of naive- and memory-phenotype T cells. J Exp Med 179: 1127–1135 8145034 10.1084/jem.179.4.1127 PMC2191431

[106] Kong FK, Chen CL, Six A, Hockett RD, Cooper MD (1999) T cell receptor gene deletion circles identify recent thymic emigrants in the peripheral T cell pool. Proc Natl Acad Sci U S A 96: 1536–1540 9990059 10.1073/pnas.96.4.1536 PMC15507

[107] Douek DC, McFarland RD, Keiser PH, Gage EA, Massey JM, et al. (1998) Changes in thymic function with age and during the treatment of HIV infection. Nature 396: 690–695 9872319 10.1038/25374

[108] Hazenberg MD, Verschuren MC, Hamann D, Miedema F, van Dongen JJ (2001) T cell receptor excision circles as markers for recent thymic emigrants: basic aspects, technical approach, and guidelines for interpretation. J Mol Med (Berl) 79: 631–640 11715066 10.1007/s001090100271

[109] Hazenberg MD, Borghans JA, de Boer RJ, Miedema F (2003) Thymic output: a bad TREC record. Nat Immunol 4: 97–99 12555089 10.1038/ni0203-97

[110] Haines CJ, Giffon TD, Lu LS, Lu X, Tessier-Lavigne M, et al. (2009) Human CD4+ T cell recent thymic emigrants are identified by protein tyrosine kinase 7 and have reduced immune function. J Exp Med 206: 275–285 19171767 10.1084/jem.20080996 PMC2646563

[111] Kohler S, Thiel A (2009) Life after the thymus: CD31+ and CD31- human naive CD4+ T-cell subsets. Blood 113: 769–774 18583570 10.1182/blood-2008-02-139154

[112] McHeyzer-Williams LJ, McHeyzer-Williams MG (2004) Developmentally distinct Th cells control plasma cell production in vivo. Immunity 20: 231–242 14975244 10.1016/s1074-7613(04)00028-7

[113] Johansson-Lindbom B, Svensson M, Wurbel MA, Malissen B, Marquez G, et al. (2003) Selective generation of gut tropic T cells in gut-associated lymphoid tissue (GALT): requirement for GALT dendritic cells and adjuvant. J Exp Med 198: 963–969 12963696 10.1084/jem.20031244 PMC2194196

[114] Chang JF, Thomas CA 3rd, Kung JT (1991) Induction of high level IL-2 production in CD4 + 8- T helper lymphocytes requires post-thymic development. J Immunol 147: 851–859 1677670

[115] Hendricks DW, Fink PJ (2011) Recent thymic emigrants are biased against the T-helper type 1 and toward the T-helper type 2 effector lineage. Blood 117: 1239–1249 21048154 10.1182/blood-2010-07-299263 PMC3056472

[116] Clise-Dwyer K, Huston GE, Buck AL, Duso DK, Swain SL (2007) Environmental and intrinsic factors lead to antigen unresponsiveness in CD4(+) recent thymic emigrants from aged mice. J Immunol 178: 1321–1331 17237378 10.4049/jimmunol.178.3.1321

[117] Rose S, Lichtenheld M, Foote MR, Adkins B (2007) Murine neonatal CD4+ cells are poised for rapid Th2 effector-like function. J Immunol 178: 2667–2678 17312108 10.4049/jimmunol.178.5.2667 PMC2112939

[118] Priyadharshini B, Welsh RM, Greiner DL, Gerstein RM, Brehm MA (2010) Maturation-dependent licensing of naive T cells for rapid TNF production. PLoS One 5: e15038 21124839 10.1371/journal.pone.0015038 PMC2991336

[119] Makaroff LE, Hendricks DW, Niec RE, Fink PJ (2009) Postthymic maturation influences the CD8 T cell response to antigen. Proc Natl Acad Sci U S A 106: 4799–4804 19270077 10.1073/pnas.0812354106 PMC2660772

[120] Joshi NS, Kaech SM (2008) Effector CD8 T cell development: a balancing act between memory cell potential and terminal differentiation. J Immunol 180: 1309–1315 18209024 10.4049/jimmunol.180.3.1309

[121] Houston EG Jr, Boursalian TE, Fink PJ (2012) Homeostatic signals do not drive post-thymic T cell maturation. Cell Immunol 274: 39–45 22398309 10.1016/j.cellimm.2012.02.005 PMC3334402

[122] Hsu FC, Pajerowski AG, Nelson-Holte M, Sundsbak R, Shapiro VS (2011) NKAP is required for T cell maturation and acquisition of functional competency. J Exp Med 208: 1291–1304 21624937 10.1084/jem.20101874 PMC3173250

[123] Kyewski B, Klein L (2006) A central role for central tolerance. Annu Rev Immunol 24: 571–606 16551260 10.1146/annurev.immunol.23.021704.115601

[124] Yin Y, Li Y, Kerzic MC, Martin R, Mariuzza RA (2011) Structure of a TCR with high affinity for self-antigen reveals basis for escape from negative selection. EMBO J 30: 1137–1148 21297580 10.1038/emboj.2011.21 PMC3061028

[125] Houston EG. Jr, Fink PJ (2009) MHC drives TCR repertoire shaping, but not maturation, in recent thymic emigrants. J Immunol 183: 7244–7249 19915060 10.4049/jimmunol.0902313 PMC2782759

[126] Nishio J, Suzuki M, Nanki T, Miyasaka N, Kohsaka H (2004) Development of TCRB CDR3 length repertoire of human T lymphocytes. Int Immunol 16: 423–431 14978016 10.1093/intimm/dxh046

[127] Matsutani T, Ohmori T, Ogata M, Soga H, Kasahara S, et al. (2007) Comparison of CDR3 length among thymocyte subpopulations: impacts of MHC and BV segment on the CDR3 shortening. Mol Immunol 44: 2378–2387 17156844 10.1016/j.molimm.2006.10.026

[128] Alferink J, Tafuri A, Vestweber D, Hallmann R, Hammerling GJ, et al. (1998) Control of neonatal tolerance to tissue antigens by peripheral T cell trafficking. Science 282: 1338–1341 9812902 10.1126/science.282.5392.1338

[129] Lukacs-Kornek V, Turley SJ (2011) Self-antigen presentation by dendritic cells and lymphoid stroma and its implications for autoimmunity. Curr Opin Immunol 23: 138–145 21168318 10.1016/j.coi.2010.11.012 PMC3042528

[130] Gardner JM, Devoss JJ, Friedman RS, Wong DJ, Tan YX, et al. (2008) Deletional tolerance mediated by extrathymic Aire-expressing cells. Science 321: 843–847 18687966 10.1126/science.1159407 PMC2532844

[131] Thangavelu G, Parkman JC, Ewen CL, Uwiera RR, Baldwin TA, et al. (2011) Programmed death-1 is required for systemic self-tolerance in newly generated T cells during the establishment of immune homeostasis. J Autoimmun 36: 301–312 21441014 10.1016/j.jaut.2011.02.009

[132] Elgbratt K, Kurlberg G, Hahn-Zohric M, Hornquist EH (2010) Rapid migration of thymic emigrants to the colonic mucosa in ulcerative colitis patients. Clin Exp Immunol 162: 325–336 20840654 10.1111/j.1365-2249.2010.04230.x PMC2996600

[133] Li Y, Geng S, Yin Q, Chen S, Yang L, et al. (2010) Decreased level of recent thymic emigrants in CD4+ and CD8 + T cells from CML patients. J Transl Med 8: 47 20470401 10.1186/1479-5876-8-47 PMC2880023

